# Supplementary figures and images for: wnt16 regulates spine and muscle morphogenesis through parallel signals from notochord and dermomyotome
Source: PLoS Genet. 2022 Nov 8;18(11):e1010496. doi: 10.1371/journal.pgen.1010496 (PMC9674140; doi:10.1371/journal.pgen.1010496)

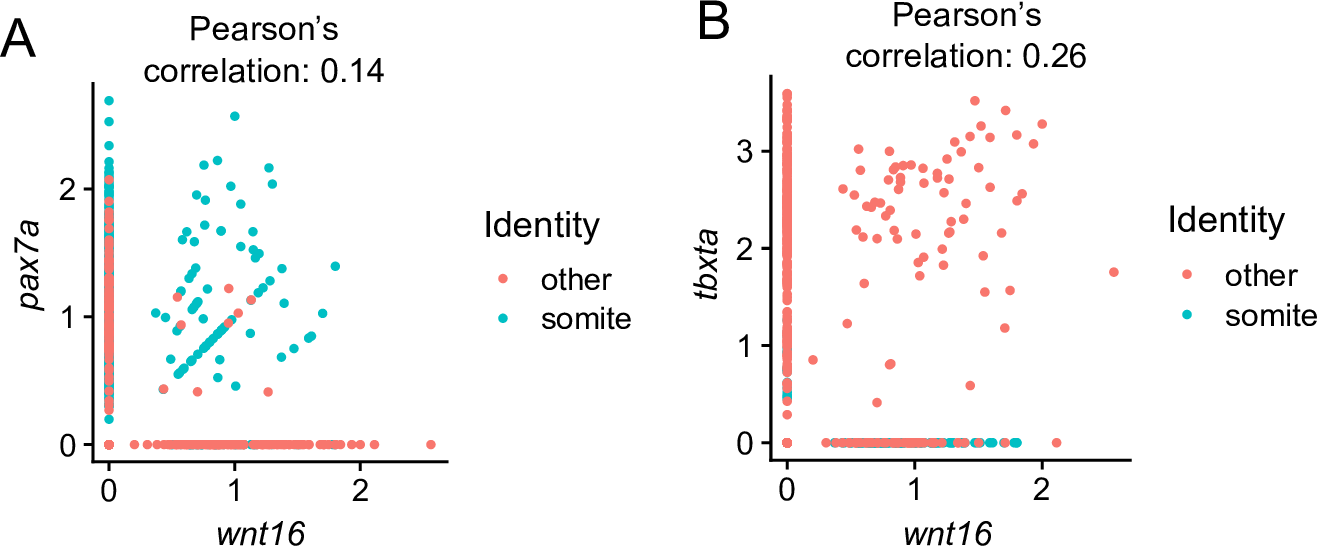

Supplement: S1 Fig — (TIF) [file pgen.1010496.s001.tif]

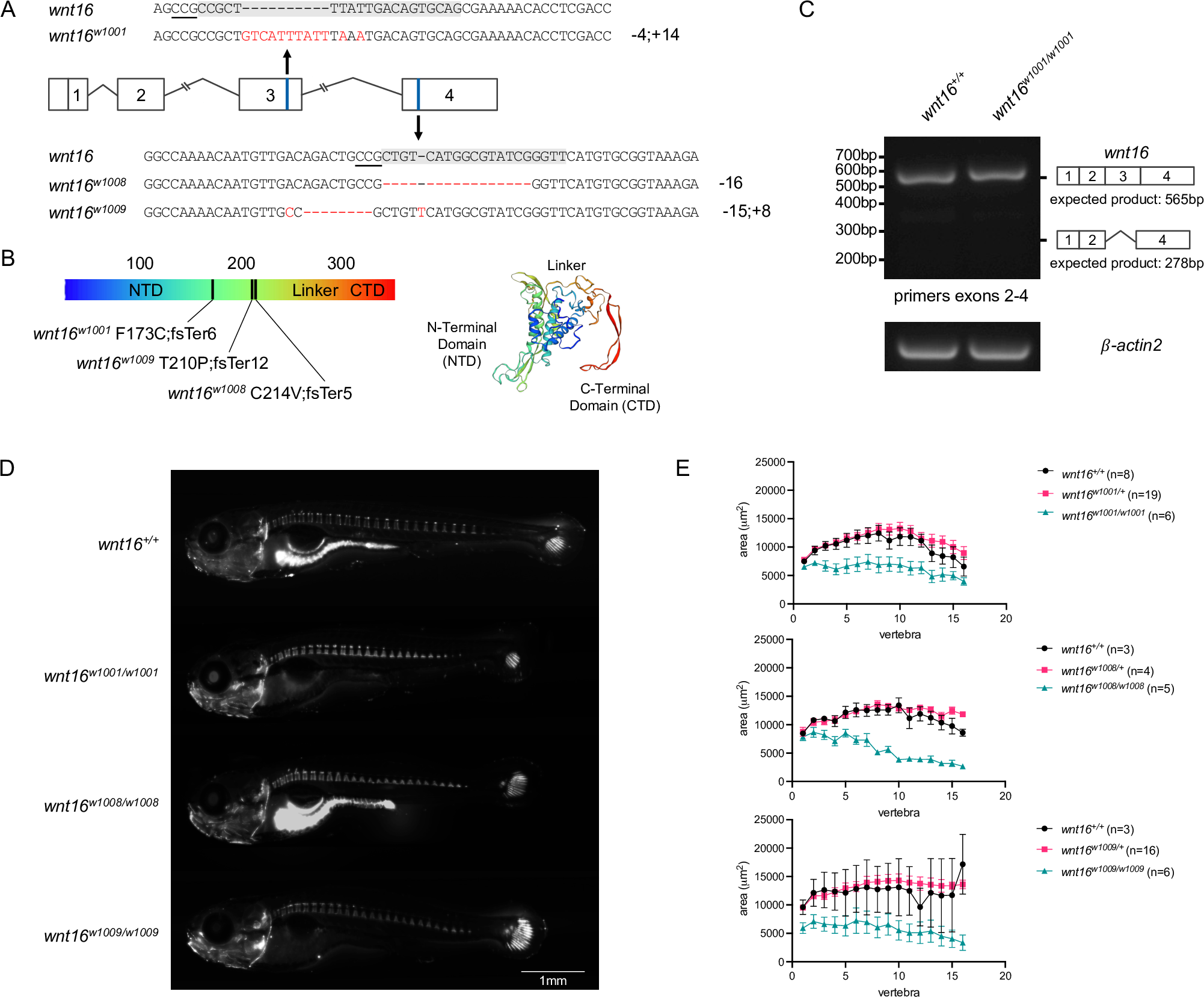

Supplement: S2 Fig — (A) Sequence and genomic location of w1001, w1008, and w1009. Grey highlight indicates gRNA target sequence used for CRISPR-based gene editing with PAM underlined. (B) Predicted effects of alleles on amino acid sequence. (C) RT-PCR assessing wnt16 transcript in wnt16w1001 mutants. No evidence of transcript reduction or alternative splicing is observed. (D) Calcein staining of 13 dpf animals show similar reductions in vertebral mineralization and post-cranial body length in w1001, w1008, and w1009 mutants. (E) Quantification of mineralized area shows similar changes in mutants for all three alleles. (TIF) [file pgen.1010496.s002.tif]

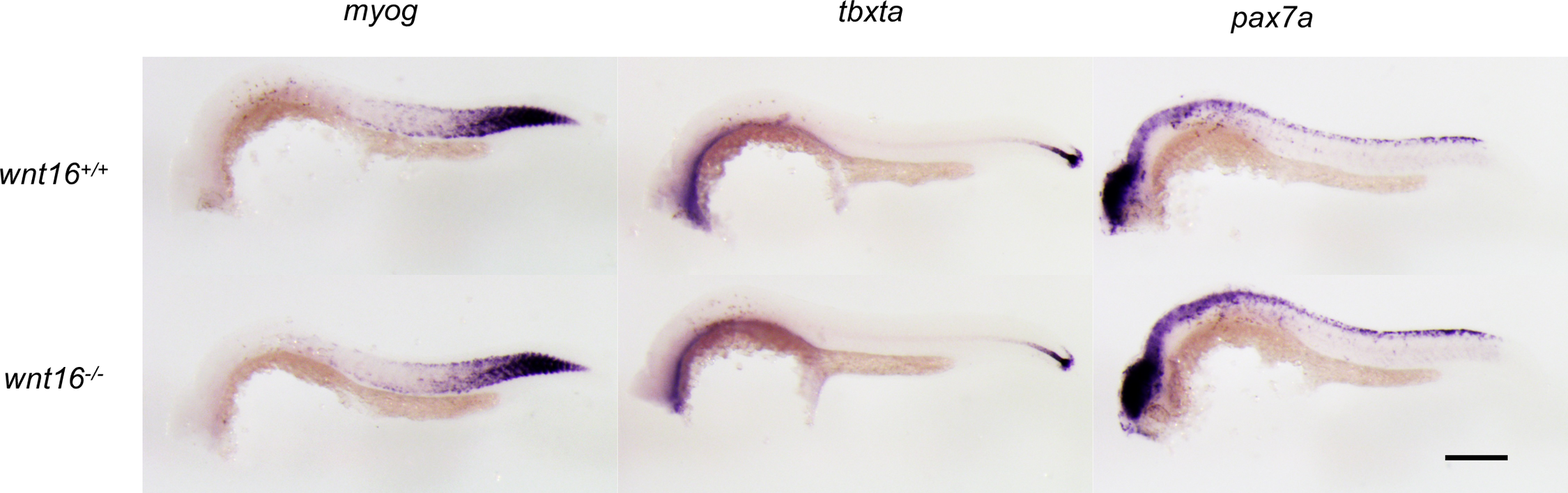

Supplement: S3 Fig — Scale bar: 200 μm. (TIF) [file pgen.1010496.s003.tif]

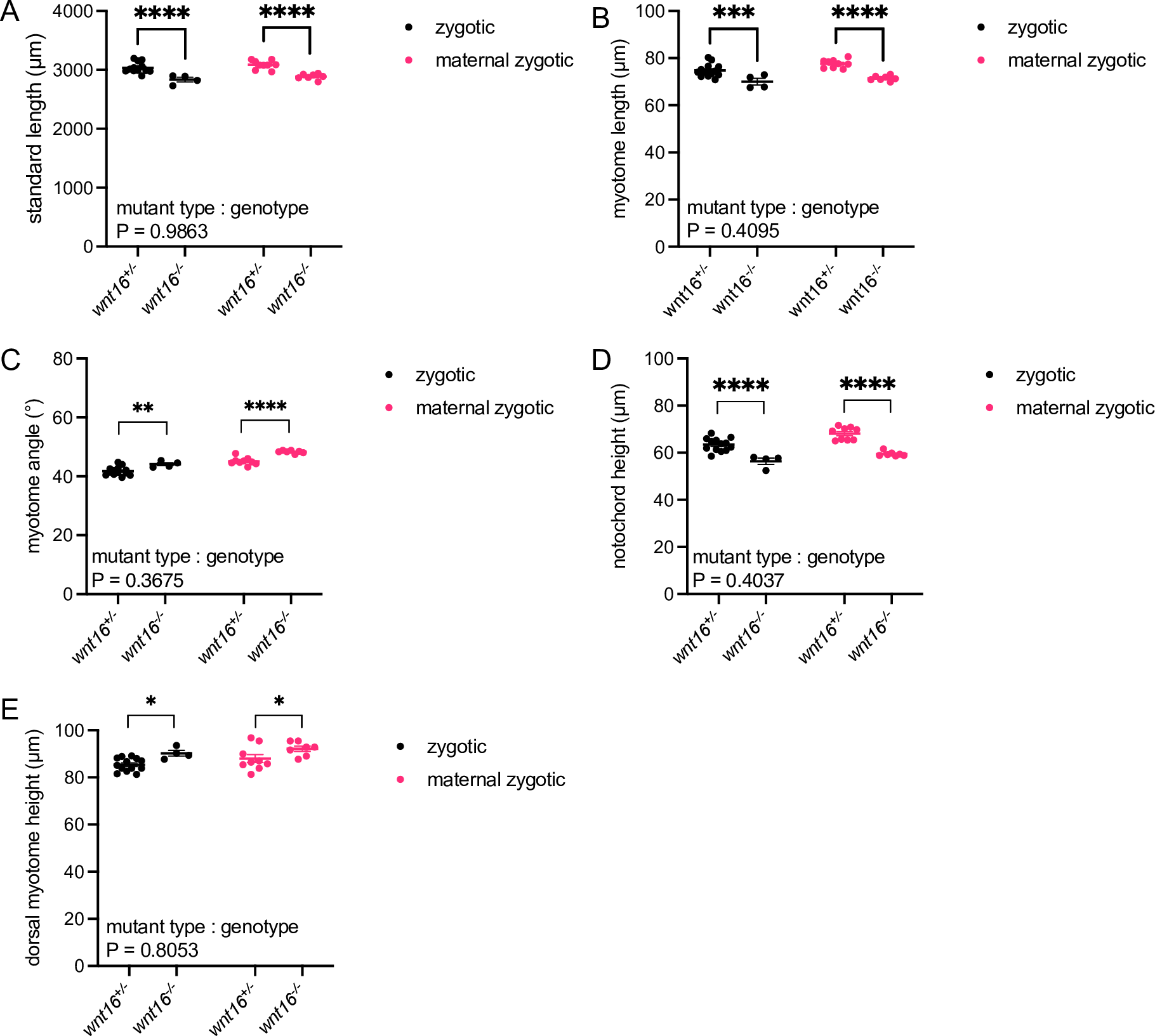

Supplement: S4 Fig — P-values were determined using a two-way ANOVA with Fisher’s LSD post hoc test. *p<0.05, **p<0.01, ***p<0.001, ****p<0.0001. (TIF) [file pgen.1010496.s004.tif]

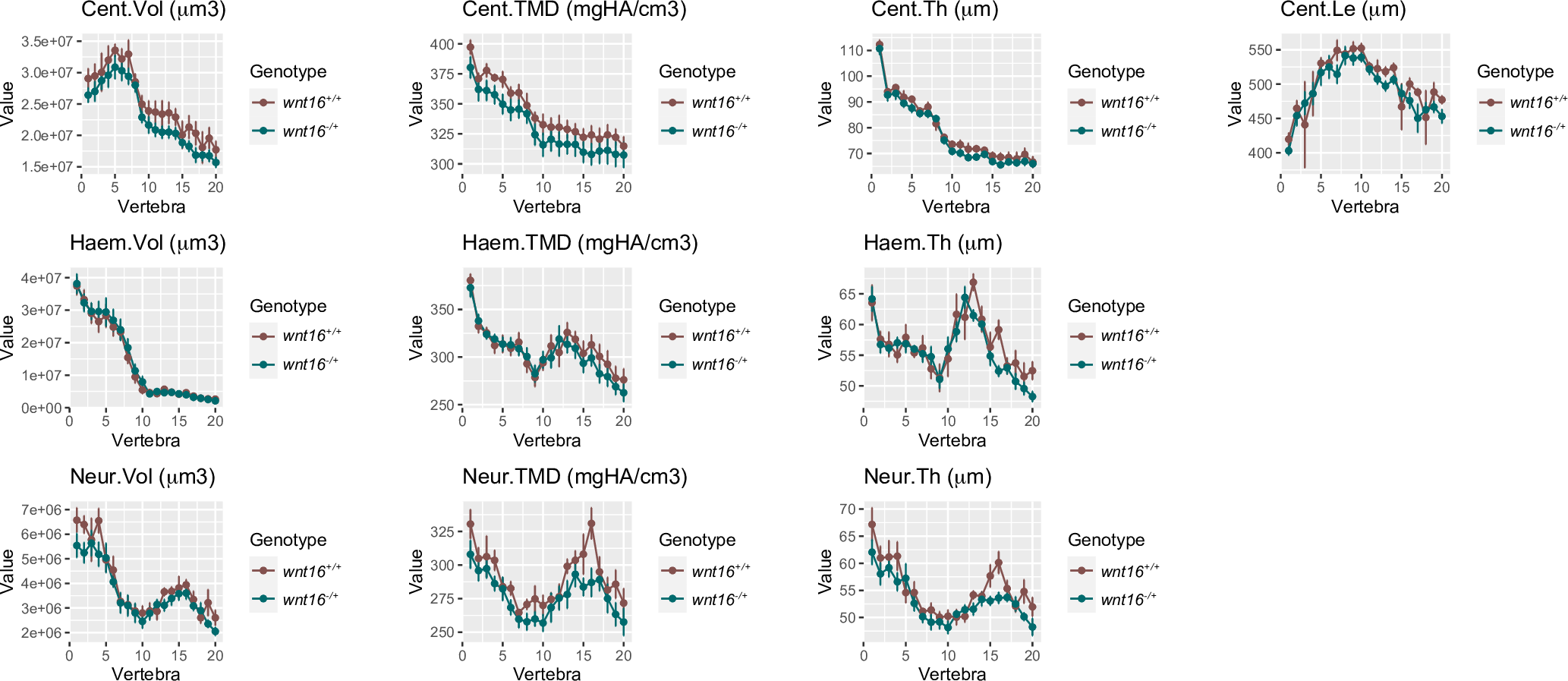

Supplement: S5 Fig — (TIF) [file pgen.1010496.s005.tif]

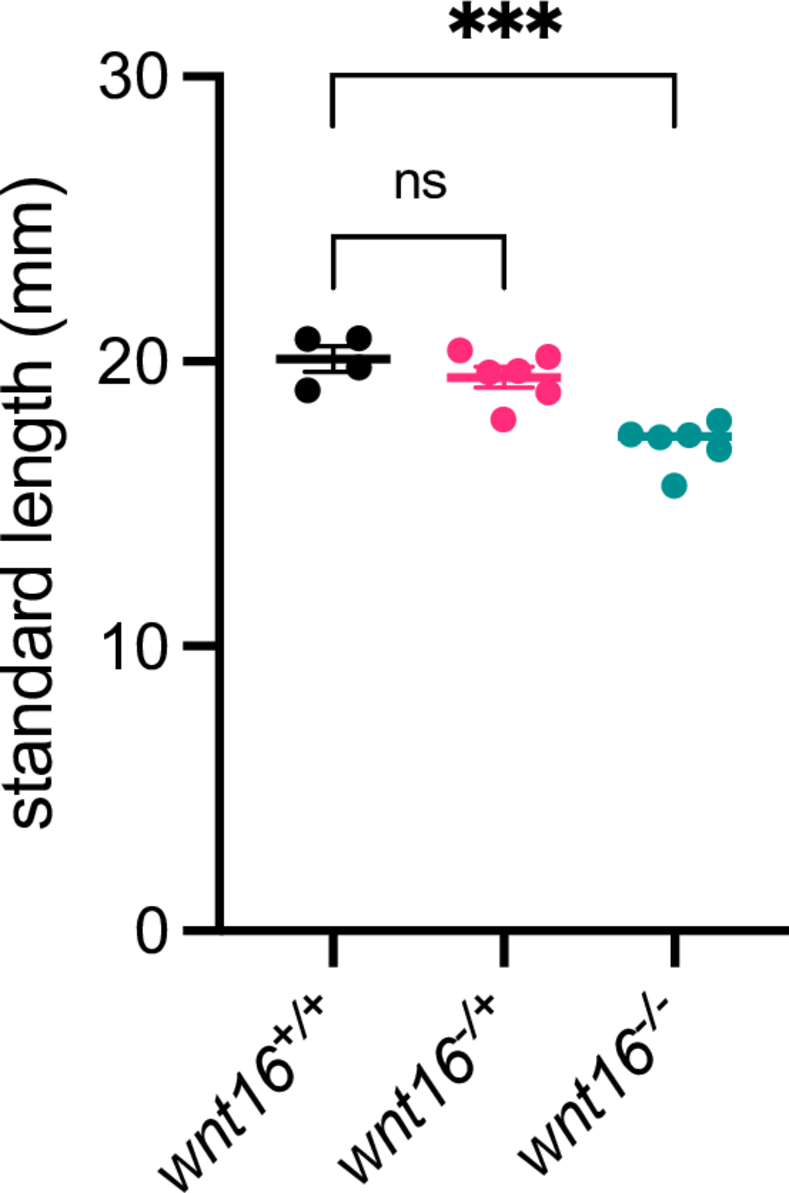

Supplement: S6 Fig — P-values were determined using one-way ANOVA with Fisher’s LSD post hoc test. ***p<0.001, ns: not significant. (TIF) [file pgen.1010496.s006.tif]

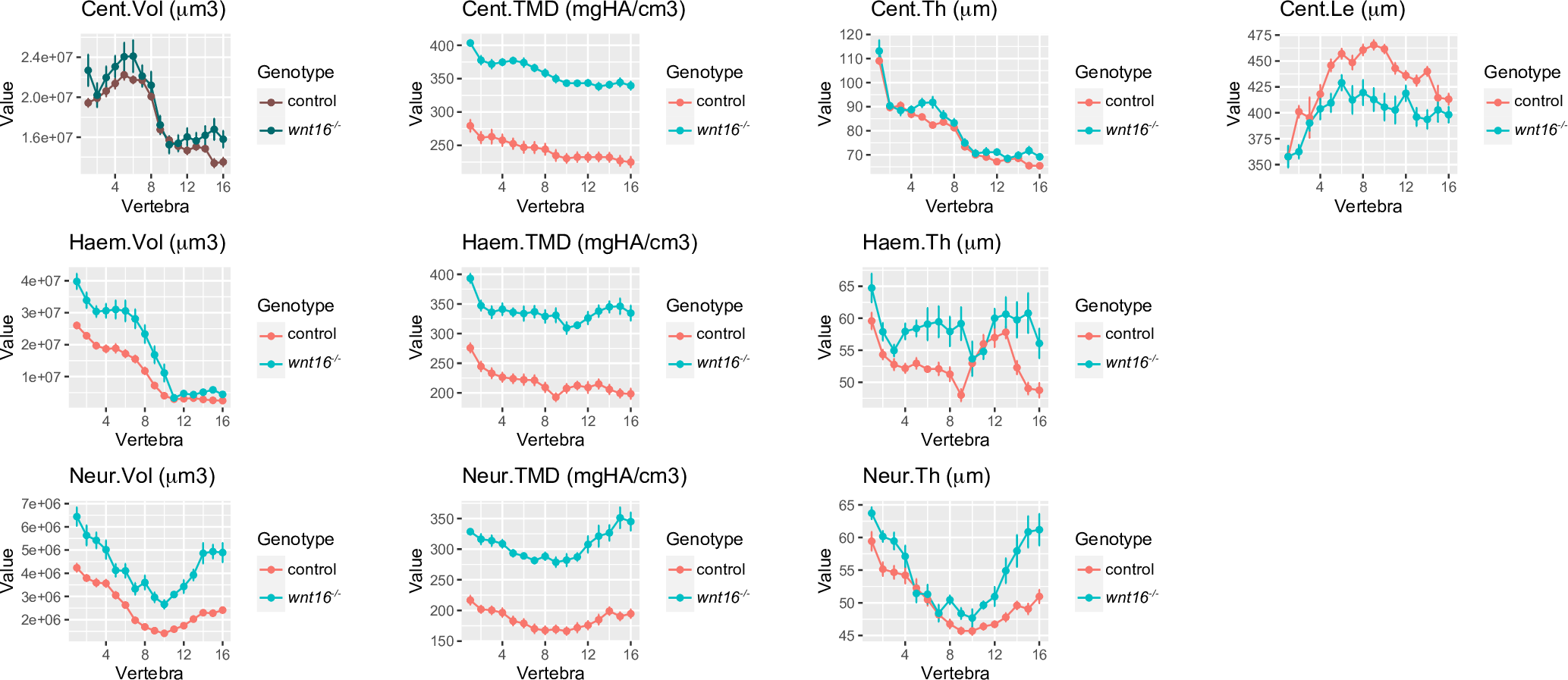

Supplement: S7 Fig — wnt16-/- mutants exhibit significant differences for most measures (all except for Cent.Vol) when normalized for differences in standard length. (TIF) [file pgen.1010496.s007.tif]

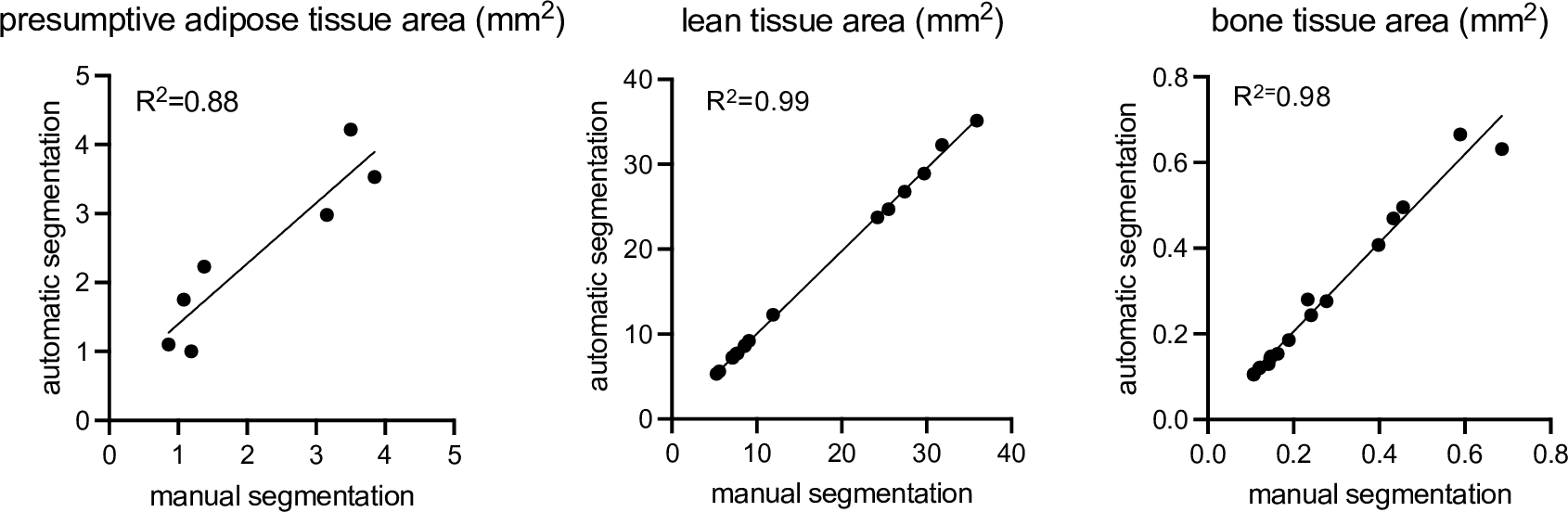

Supplement: S8 Fig — (TIF) [file pgen.1010496.s008.tif]

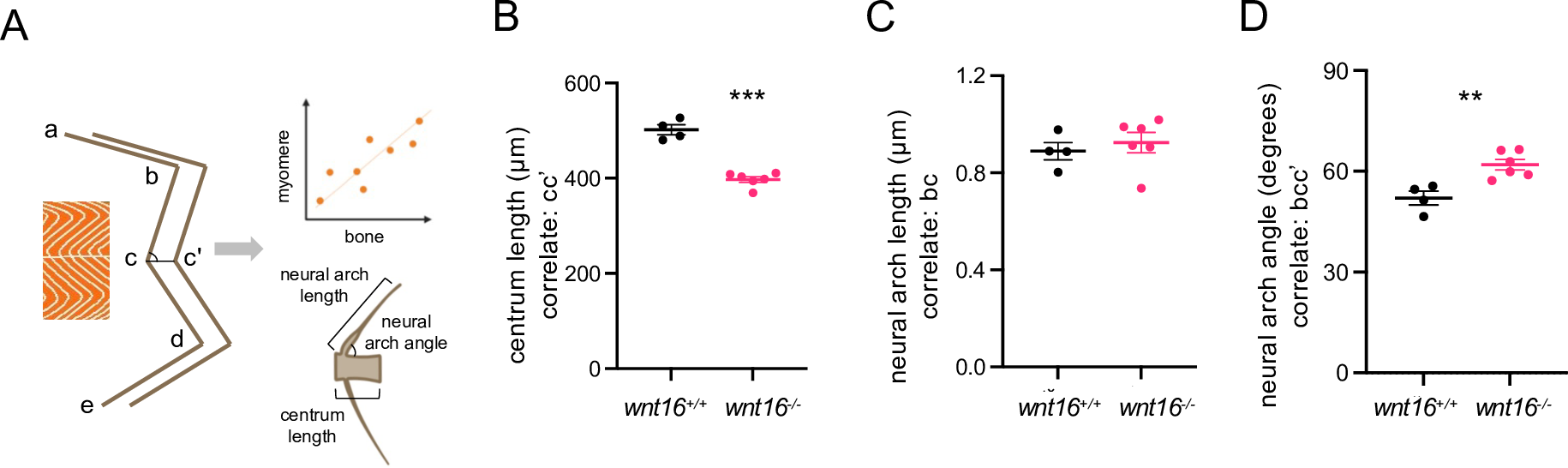

Supplement: S9 Fig — (A) Schematic demonstrating correlated features. (B-D) wnt16-/- mutants exhibit altered centrum length and neural arch angle. P-values were determined using an unpaired t-test. **p<0.01, ***p<0.001. (TIF) [file pgen.1010496.s009.tif]

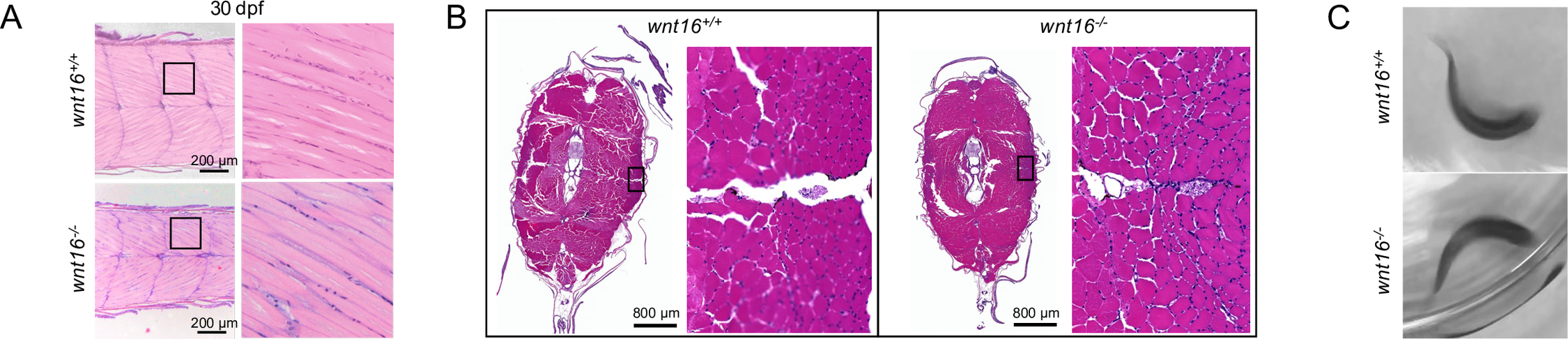

Supplement: S10 Fig — H&E-stained sections showing frontal section of the caudal region and corresponding magnified areas in 30 dpf animals (A) and transverse sections in adult animals (B). wnt16-/- mutants exhibited no obvious differences in muscle segmentation or fiber morphology. (C) Mutants and wildtype clutchmates exhibited full-length body flexions when the startle-induced C-start response was evoked. (TIF) [file pgen.1010496.s010.tif]

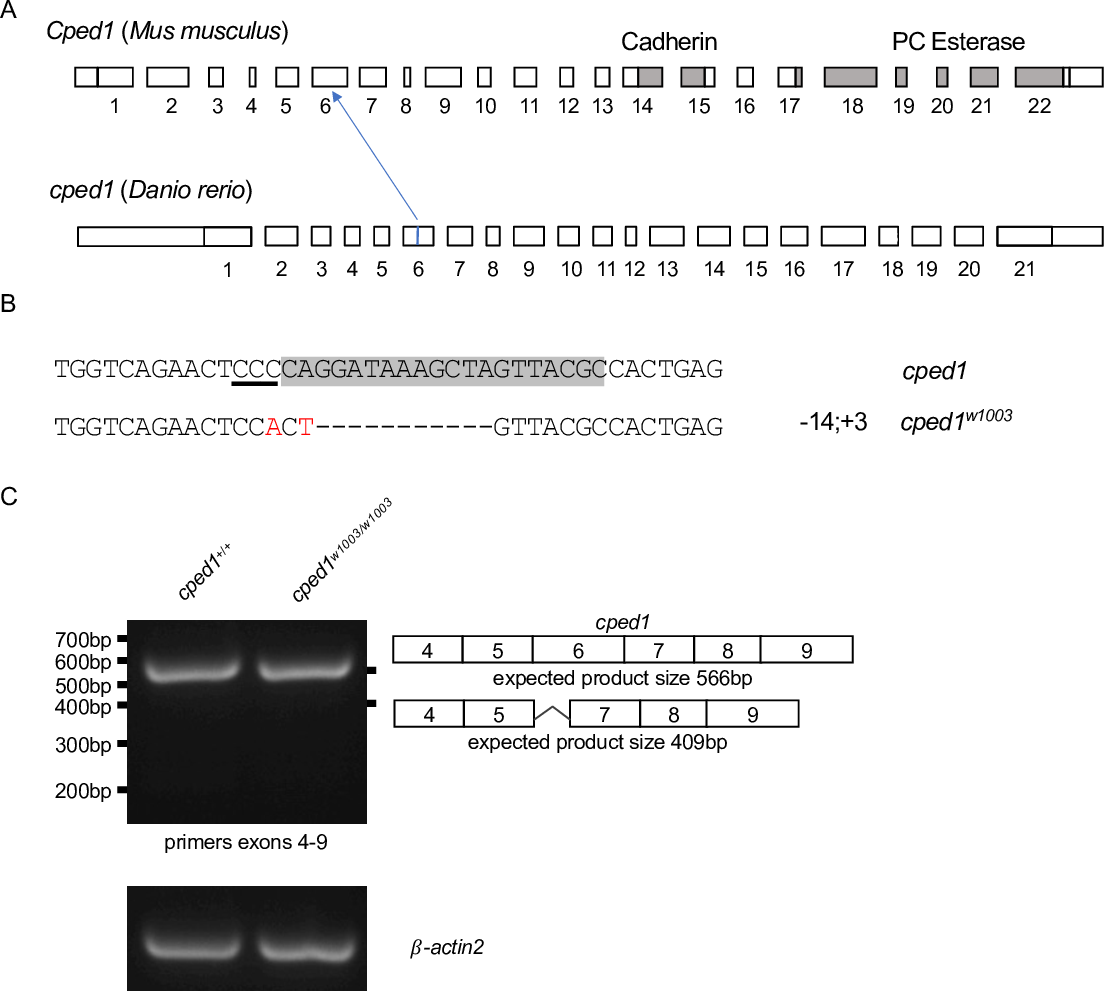

Supplement: S11 Fig — (A) Genomic location of w1003 and its corresponding location mapped to mouse Cped1. Locations of Cadherin and PC Esterase domains in mouse Cped1 are from [49]. (B) Genomic sequence of w1003. Grey highlight indicates gRNA target sequence used for CRISPR-based gene editing with PAM underlined. (C) RT-PCR assessing cped1 transcript in cped1w1003 mutants. No evidence of transcript reduction or alternative splicing is observed. (TIF) [file pgen.1010496.s011.tif]

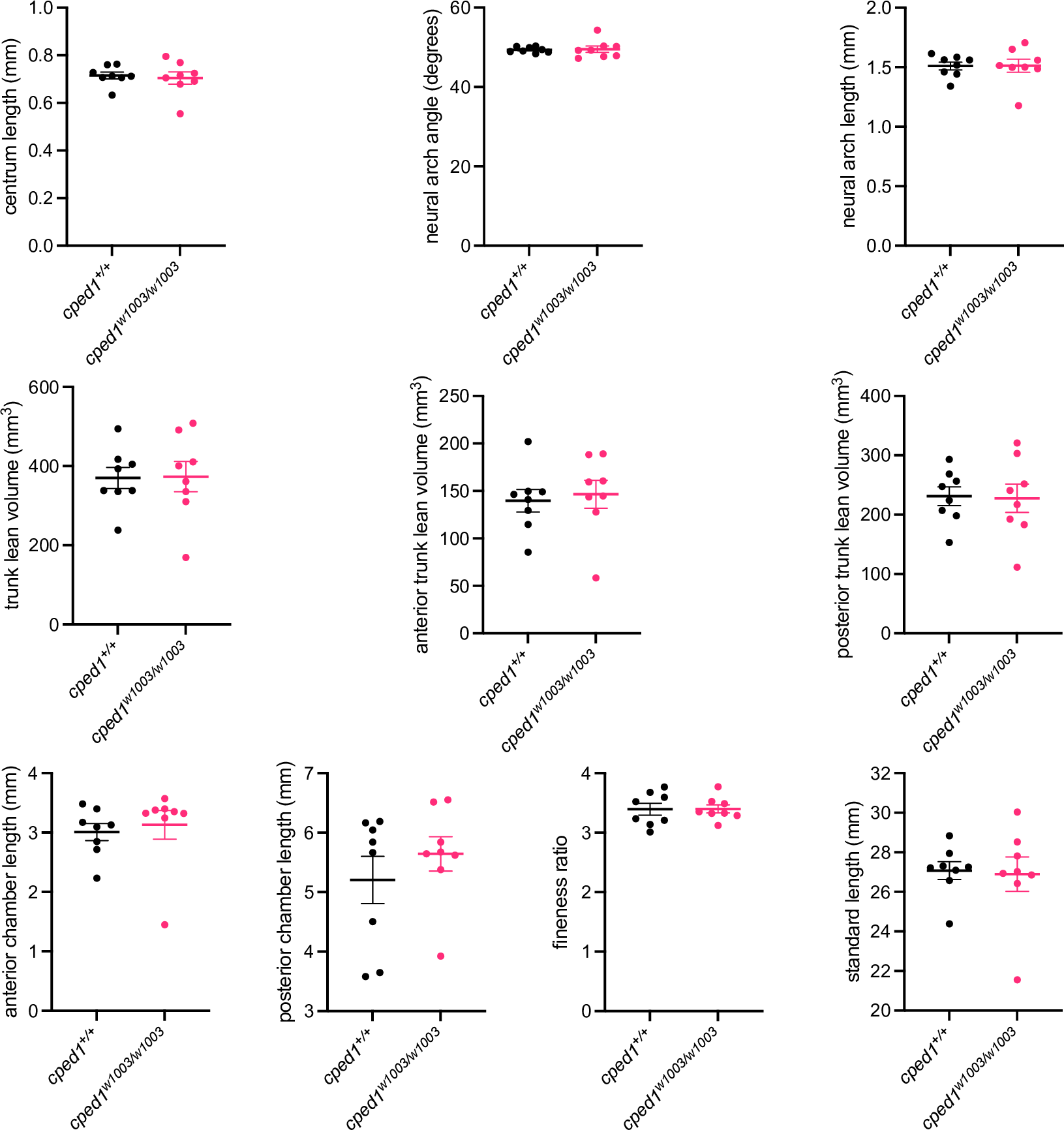

Supplement: S12 Fig — (TIF) [file pgen.1010496.s012.tif]
