# Supplementary material for: wnt16 regulates spine and muscle morphogenesis through parallel signals from notochord and dermomyotome
Source: PLoS Genet. 2022 Nov 8;18(11):e1010496. doi: 10.1371/journal.pgen.1010496 (PMC9674140; doi:10.1371/journal.pgen.1010496)
Supplement: S1 Code — (DOCX) [file pgen.1010496.s013.docx]

**Sample MATLAB code used for computing lean tissue volume**

v = dicomreadVolume('mutant 01');

v = squeeze(v);

thresh1 = 0;

thresh2 = 1043;

thresh3 = 6368;

startk = 236;

endk = 822;

dims = size(v);

imax = dims(1);

jmax = dims(2);

kmax = dims(3);

% Target A = noise less than 0

targetA = v;

% Target B = noise between 0 and soft tissue

targetB = v;

% Target C = soft tissue ie muscle

targetC = v;

% Target D = bone

targetD = v;

for i = 1:imax

for j = 1:jmax

for k = 1:kmax

p = v(i, j, k);

%target A

if(p < thresh1)

targetA(i, j, k) = 1;

else

targetA(i, j, k) = 0;

end

%target B

if((p > thresh1) && (p < thresh2))

targetB(i, j, k) = 1;

else

targetB(i, j, k) = 0;

end

%target C

if((p > thresh2) && (p < thresh3))

targetC(i, j, k) = 1;

else

targetC(i, j, k) = 0;

end

%target D

if(p > thresh3)

targetD(i, j, k) = 1;

else

targetD(i, j, k) = 0;

end

end

end

end

%cross section

csA(k) = 0;

csB(k) = 0;

csC(k) = 0;

csD(k) = 0;

% output is in mm^2

for k = 1:kmax

csA(k) = sum(targetA(:,:,k), 'all') * 0.000441;

csB(k) = sum(targetB(:,:,k), 'all') * 0.000441;

csC(k) = sum(targetC(:,:,k), 'all') * 0.000441;

csD(k) = sum(targetD(:,:,k), 'all') * 0.000441;

end

% output is in mm^3

volA = (sum(csA) * 0.021);

volB = (sum(csB) * 0.021);

volC = (sum(csC) * 0.021);

volD = (sum(csD) * 0.021);

headlesscsA = csA(startk:endk);

headlesscsB = csB(startk:endk);

headlesscsC = csC(startk:endk);

headlesscsD = csD(startk:endk);

% output is in mm^3

headlessvolC = (sum(headlesscsC) * 0.021);

headlessvolD = (sum(headlesscsD) * 0.021);
