# Supplementary material for: wnt16 regulates spine and muscle morphogenesis through parallel signals from notochord and dermomyotome
Source: PLoS Genet. 2022 Nov 8;18(11):e1010496. doi: 10.1371/journal.pgen.1010496 (PMC9674140; doi:10.1371/journal.pgen.1010496)
Supplement: Attachment — Submitted filename: WNT16 Paper II v14 100422 Response to Reviewers.docx [file pgen.1010496.s015.docx]

**RESPONSE TO REVIEWERS**

**Reviewer 1**

**1. The section describing figure 4 (starting with ‘Next, we examined whether wnt16 contributes to specific myotome compartments or developmental axes’) is duplicated. I would suggest keeping the second version.**

This has been corrected.

**2. Figure 6, quantification of mineralized domain morphology. Please add how many fish and how many vertebrae per fish were analyzed in C-G. Which vertebrae were analyzed?**

In the caption for Figure 6 we have clarified how many fish and how many vertebrae per fish were analyzed in C-G, as well as which vertebrae were analyzed.

**3. Figure 8B, it is unclear what ‘a’ refers to. Is this the length or the posterior height of the fish?**

In Figure 8B we have clarified in the schematic that ‘a’ refers to the length of the fish.

**4. Figure 9A, the gene names are unreadable**

We have increased the size of the gene names in Figure 9A.
